# Mummichog gill and operculum exhibit functionally consistent *claudin-10* paralog profiles and Claudin-10c hypersaline response

**DOI:** 10.1242/bio.058868

**Published:** 2021-07-26

**Authors:** Chun Chih Chen, William S. Marshall, George N. Robertson, Regina R. F. Cozzi, Scott P. Kelly

**Affiliations:** 1 Department of Biology, York University, Toronto, ON M3J 1P3, Canada; 2 Department of Biology, St. Francis Xavier University, Antigonish, NS B2G 2W5, Canada

**Keywords:** Osmoregulation, Tight junction, *Fundulus*, Ionocyte, Accessory cell, CFTR

## Abstract

Claudin (Cldn)-10 tight junction (TJ) proteins are hypothesized to form the paracellular Na^+^ secretion pathway of hyposmoregulating mummichog (*Fundulus heteroclitus*) branchial epithelia. Organ-specific expression profiles showed that only branchial organs [the gill and opercular epithelium (OE)] exhibited abundant *cldn-10* paralog transcripts, which typically increased following seawater (SW) to hypersaline (2SW) challenge. Post-translational properties, protein abundance, and ionocyte localization of Cldn-10c, were then examined in gill and OE. Western blot analysis revealed two Cldn-10c immunoreactive bands in the mummichog gill and OE at ∼29 kDa and ∼40 kDa. The heavier protein could be eliminated by glycosidase treatment, demonstrating the novel presence of a glycosylated Cldn-10c. Protein abundance of Cldn-10c increased in gill and OE of 2SW-exposed fish. Cldn-10c localized to the sides of gill and OE ionocyte apical crypts and partially colocalized with cystic fibrosis transmembrane conductance regulator and F-actin, consistent with TJ complex localization. Cldn-10c immunofluorescent intensity increased but localization was unaltered by 2SW conditions. In support of our hypothesis, *cldn-10*/Cldn-10 TJ protein dynamics in gill and OE of mummichogs and TJ localization are functionally consistent with the creation and maintenance of salinity-responsive, cation-selective pores that facilitate Na^+^ secretion in hyperosmotic environments.

## Abstract (precis)

**Summary:** The role of claudin-10 tight junction proteins in paracellular salt secretion across fish branchial epithelia is indicated by organ-specific responses to hyperosmotic conditions and their association with salt secreting transcellular proteins

## INTRODUCTION

Teleost fish gill and opercular epithelium (OE) contain high densities of ionocytes that are specialized to secrete Na^+^ and Cl^−^ into seawater (SW) and hypersaline environments (Edwards and Marshall, 2013; Evans et al., 2005; Marshall and Grosell, 2006). For marine teleost fishes, the secondary active accumulation of Cl^−^ into ionocytes via the basolateral Na^+^, K^+^, 2Cl^−^ and the Cl^−^ exit by passive diffusion via the apical cystic fibrosis transmembrane conductance regulator (CFTR) anion channel mediates transcellular Cl^−^ secretion into a cup-shaped apical microenvironment, the ionocyte apical crypt. In parallel, a cation-selective paracellular pathway employs a transepithelial positive electrical gradient of approximately +35-40 mV (Guggino, 1980; Marshall et al., 2017) to drive Na^+^ down its electrochemical gradient from blood to SW (Degnan and Zadunaisky, 1980; Pequeux et al., 1988). Paracellular Na^+^ transport occurs through single-stranded, cation-permeable (‘leaky’) intercellular tight junctions (TJs) that exclusively form between marine fish ionocytes and a cell that resides adjacent to the ionocyte, the accessory cell (AC) (Karnaky, 1991; Sardet et al., 1979). Although some progress has been made in terms of elucidating which specific TJ proteins establish the required cation-selective pores between ionocytes and ACs (see below), there is a great deal that remains largely unknown.

Claudin (Cldn) proteins are incorporated into intercellular TJs of vertebrate epithelia where they occupy a critically important role in the regulation of TJ permselectivity (Chasiotis et al., 2012; Krug et al., 2012, 2014). Some of these epithelial intercellular junctions are non-selective ‘leaky’ junctions, whereas others are selective to certain molecular species (e.g. cations or anions) and constitute cation or anion permeable ‘pores’ (Shen et al., 2011). Because the gill epithelium of teleost fishes changes its transport characteristics from ion uptake in fresh water (FW) to ion secretion in SW, with concomitant changes in paracellular permeability of the junctions linking ionocytes and ACs, euryhaline species have been used to study the functional dynamics of *cldn*/Cldn responses to these changing ion transport demands (for recent reviews see Chasiotis et al., 2012; Kolosov et al., 2013).

Several lines of evidence support a model involving insertion of Cldn-10 proteins to form (or contribute to the formation of) cation-selective pores between ionocytes and ACs of SW residing fishes. These include observations of restricted organ- and cell-specific *cldn-10*/Cldn-10 paralog distribution patterns, salinity-induced alterations in sub-cellular distribution as well as changes in abundance of *cldn*-10 transcript and Cldn-10 protein paralogs following salinity transfer or hormone treatment (Bossus et al., 2015; Bui and Kelly, 2014, 2015; Bui et al., 2010; Kolosov et al., 2014; Loh et al., 2004; Marshall et al., 2018; Tipsmark et al., 2008, 2009; Trubitt et al., 2015). However, further studies on salt secretory epithelia of euryhaline teleost fishes are needed to discover exactly which Cldns are involved and how they form cation-selective pores.

The mummichog (*Fundulus heteroclitus*) is an important model organism that has provided significant genomic and physiological insights into molecular and cellular mechanisms that underlie gill function (Burnett et al., 2007; Cozzi et al., 2015; Whitehead et al., 2011). This is partly because mummichogs naturally inhabit, and in a laboratory setting readily acclimate to, salinities ranging from FW to strongly hypersaline conditions for extended periods of time (Griffith, 1974), and also because early observations revealed that the OE of this species is an excellent surrogate gill model that can be used for studies that are not possible to conduct using the architecturally complex gill itself (Degnan et al., 1977; Degnan and Zadunaisky, 1980; Karnaky, 1991; Marshall et al., 1999; Marshall and Grosell, 2006). In addition, this species exhibits consistent differential regulation of transcripts encoding TJ proteins upon exposure to different salinities and diversifying selection between TJ protein transcripts in populations inhabiting different osmotic environments (see Reid et al., 2017). Therefore, the mummichog is a robust model for examining TJ modifications in response to salinity stress. To this end, we have recently examined *cldn-10c*, *-10d*, *-10e* and *-10f* mRNA abundance in mummichog gill following acclimation to increased salinity and described cation selectivity of the branchial salt secretory pore using the OE (Marshall et al., 2018). It was found that following acclimation of mummichogs from FW to SW, increased transcript abundance of *cldn-10d* and *-10e* occurred in the gill, and this was in line with salinity-induced changes of *cldn-10* paralogs in the gill tissues of other euryhaline fish species (see Marshall et al., 2018). A particularly novel observation was that acclimation of mummichogs to hypersaline conditions (i.e. from SW to 2SW) produced enhanced salt secretory activity of branchial trans- and paracellular pathways as well as a significant and sustained increase in the mRNA abundance of select *cldn-10* paralogs that had not been responsive to SW acclimation, i.e. *cldn-10c*, and *-10f* (Marshall et al., 2018). It has been reported that hypersaline stress causes further elaboration of TJ structure and enhanced ionic conductance (Cozzi et al., 2015) such that, even with a reduced transepithelial electromotive driving force, NaCl secretion can continue. Therefore, our previous work suggests that *cldn-10* paralogs in the branchial tissues of mummichogs may exhibit a functionally tiered response to elevated environmental ion levels that is dictated by how high the salinity becomes. This would make physiological sense for an organism that, under normal circumstances, will experience tidal-driven fluctuations in the salt content of its surroundings that would range between FW above and SW below a halocline, but could also find itself isolated in an evaporating finite body of water (e.g. a tide pool) where salinity can often exceed that of SW.

The aforementioned observations provide an impetus to enhance our understanding of Cldn-10 TJ proteins in the mummichog, with an emphasis on several areas. First, despite the broadly acknowledged suitability of the OE as an appropriate surrogate gill model to study mechanisms of ion transport across the SW fish gill, no study has profiled and examined TJ proteins in the OE. The OE is a flat epithelial sheet on the underside of bony opercula that cover the branchial cavity of fishes, but it is not part of the gill per se. Therefore, despite possessing ion transport characteristics and a cellular composition which mimics the gill, the OE could be viewed as skin, and the skin of fishes possesses a different TJ protein profile than the gill (see Kolosov et al., 2013). Therefore, the molecular physiology of OE TJs should be examined to show that they contribute to salt secretion using the same TJ proteins as the gill epithelium. Next, observations of mummichog gill and OE Cldn-10 TJ proteins should extend beyond the transcriptional level and include studies that examine changes in protein abundance as well as investigate protein localization in both the gill and OE. To address these gaps in our knowledge, the current work detailed *cldn-10* paralog expression profiles across organs of the mummichog and compared organ-specific *cldn-10* paralog mRNA abundance following acclimation of fish from SW to 2SW. Based on our observations, a custom synthesized antibody for mummichog Cldn-10c was produced to provide evidence of TJ formation and function in gill and OE. We examined Cldn-10c (1) post-translational characteristics, particularly O-glycosylation of the protein associated with TJ formation and function, (2) protein abundance in response to hypersaline (2SW) conditions, and (3) immunolocalization with reference to a transcellular ion transport protein (i.e. CFTR), that is associated with ‘leaky’ cation-permeable TJs, as well as actin (JLA20).

## RESULTS

### Organ-specific distribution of *cldn-10* paralogs and salinity response

**Fig. 1.**
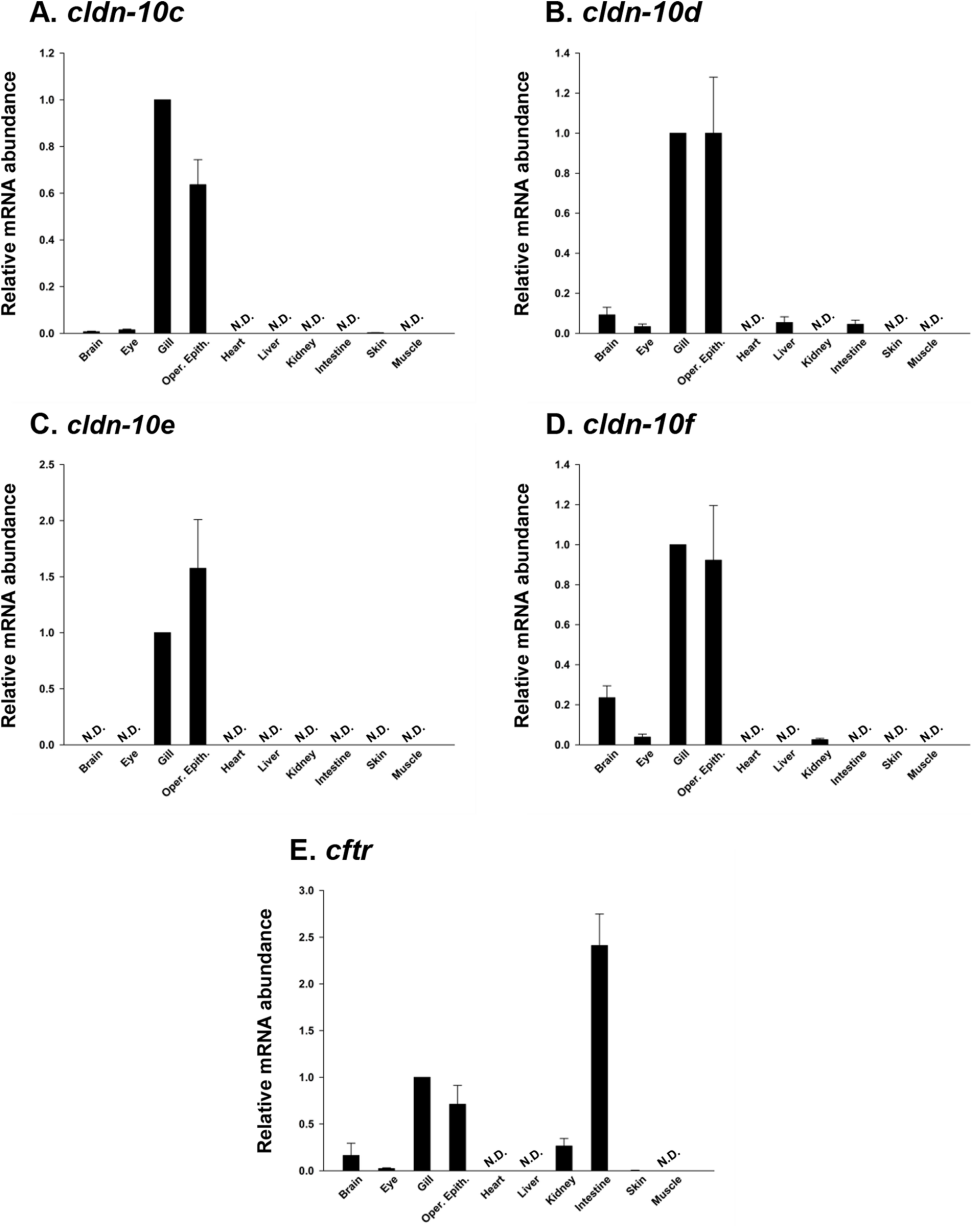
**Organ-specific expression of claudin (*cldn*) (A) *-10c*, (B) *-10d*, (C) *-10e*, (D) *-10f*, and (E) cystic fibrosis transmembrane conductance regulator (*cftr*) in adult mummichogs acclimated to seawater.** Transcript abundance was normalized using 18S RNA and expressed relative to the gill (assigned a value of 1.0) as a reference organ. All data expressed as mean values±s.e.m. (*n*=5). N.D., transcript not detected.

Examination of various mummichog organs, as well as flank muscle revealed that all *cldn-10* paralogs were predominantly expressed in the gill and OE, irrespective of their presence or absence in other organs (Fig. 1A–D). Notably, *cldn-10e* was found exclusively in the gill and OE but in no other organ/organ biopsy examined in this study. Beyond the gill and OE, *cldn-10c*, *-10d* and *-10f* transcripts were expressed in the brain and eye, but transcript abundance was low in these organs (Fig. 1A–D). All *cldn-10* paralogs were absent from the heart and muscle (Fig. 1A–D). In other organs composed mainly of epithelial tissue (i.e. liver, kidney, posterior intestine, and skin), no *cldn-10* paralog was found to be present in all. That is, no *cldn-10* paralog was broadly distributed across these organs. Instead, individual select *cldn-10* paralog mRNA was found in each organ. Specifically, *cldn-10c* was found in skin (Fig. 1A), *cldn-10d* in liver as well as posterior intestine (Fig. 1B) and *cldn-10f* in kidney (Fig. 1D), all at low levels relative to gill and OE. The distribution of *cftr* mRNA was broader than most *cldn-10* paralogs, but similar in that it was found to be quite dominant in the gill and OE (Fig. 1E). However, *cftr* mRNA of fish residing in SW was most abundant in the posterior intestine (Fig. 1E).

**Fig. 2.**
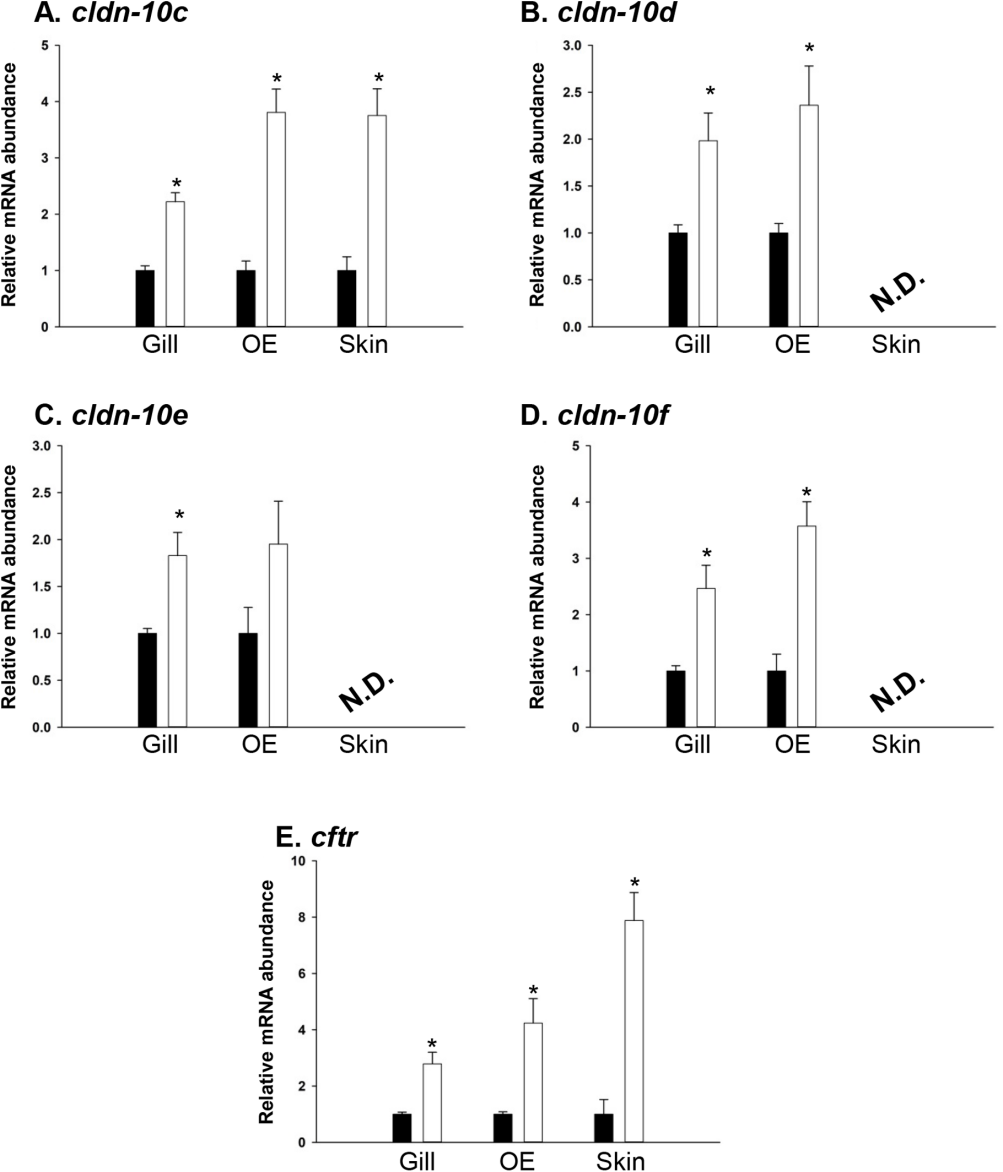
**Transcript abundance of claudin (*cldn*) (A) *-10c*, (B) *-10d*, (C) *-10e*, (D) *-10f*, and (E) cystic fibrosis transmembrane conductance regulator (*cftr*) in mummichog gill, opercular epithelium (OE), and skin following acclimation of animals from seawater (SW; black bars) to hypersaline conditions (2SW; open bars).** Transcript abundance was normalized using 18S RNA in the target tissue and 2SW transcript abundance in each organ was expressed relative SW assigned a value of 1. All data are expressed as mean values±s.e.m. (*n*=5). An asterisk (*) denotes a significant difference between the SW and 2SW group as determined using a Student's *t*-test (*P*≤0.05). N.D., transcript not detected.

Following acclimation of fish from SW to 2SW, a significant increase in gill *cldn-10c* (*P*<0.01), *-10d* (*P*=0.024), *-10e* (*P*=0.049) and *-10f* (*P*<0.01) mRNA abundance occurred (Fig. 2A–D). In the OE of fish acclimated to 2SW, *cldn-10c* (*P*<0.01), *-10d* (*P*=0.05) and *-10f* (*P*<0.01) mRNA abundance significantly increased while and *-10e* (*P*=0.20) was not significantly different (Fig. 2A–D). Transcript abundance of *cldn-10c* was also observed to significantly increase (*P*<0.01) in the skin of fish acclimated from SW to 2SW (Fig. 2A), but when present, *cldn-10* paralog mRNAs in all other organs were not found to alter in response to salinity (Fig. S1). Acclimation of fish from SW to 2SW also elicited a significant increase in gill, OE, and skin *cftr* mRNA abundance (all *P*<0.01; Fig. 2E), but no salinity-induced change in *cftr* mRNA abundance was found to occur in other organs (Fig. S1).

### Cldn-10c abundance in the gill and OE

**Fig. 3.**
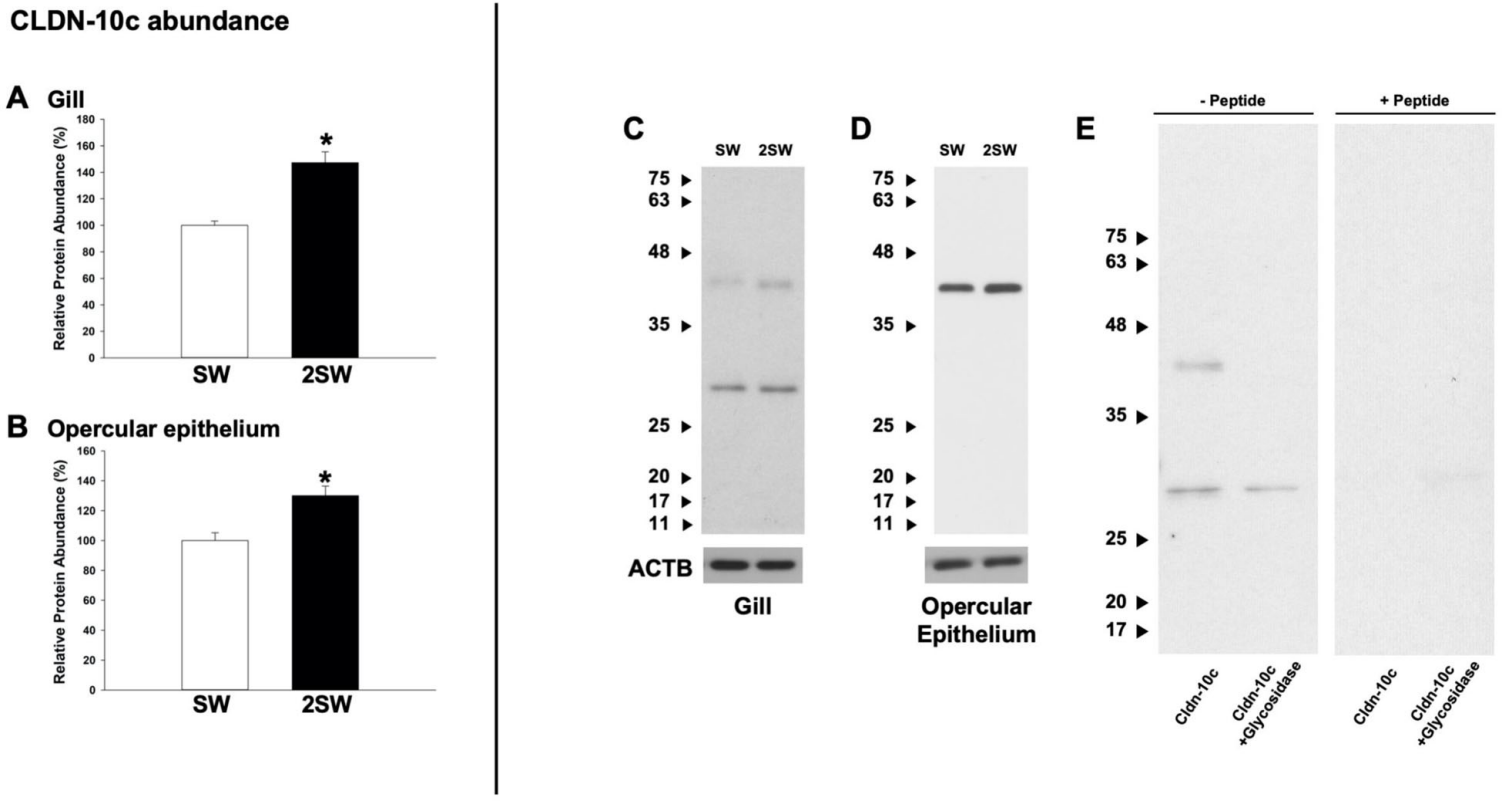
**Protein abundance of claudin-10c (Cldn-10c) in (A) gill and (B) opercular epithelium of mummichogs acclimated to seawater (SW) or hypersaline (2SW) conditions.** Panel C shows a Cldn-10c immunoblot using gill from SW and 2SW fish where two distinct bands at ∼29 and ∼40 kDa are apparent. In panel (D), the opercular epithelium (OE) reveals a singular ∼40 kDa band. Panel (E) reveals that glycosidase treatment of gill samples results in the disappearance of the ∼40 kDa immunoreactive band, and peptide blocking assay (+Peptide) results in an absence of anti-Cldn-10c immunoreactivity. Data in panels A and B are expressed as mean values±s.e.m. (*n*=5). An asterisk (*) denotes a significant difference between the SW and 2SW group as determined using a Student's *t*-test (*P*≤0.05). ACTB, actin.

Salinity acclimation from SW to 2SW resulted in a significant increase in Cldn-10c abundance in both the gill (Fig. 3A; Fig. S2) and OE (Fig. 3B; Fig. S2). Immunoblot of gill tissues showed two distinct bands with MWs of ∼29 kDa and ∼40 kDa, with the predicted MW of the Cldn-10c sequence at 29 kDa (XP_012728690). When considering the prominent 29 kDa bands in the gill, immunoblot showed a significant increase in 29 kDa band intensity in 2SW (126.76±7.49%) when compared to SW (100±5.16%; *P*<0.01; *n*=5). For the 40 kDa band in gill, intensity in 2SW was 157.54±9.57% compared to SW at 100±6.80% (*P*<0.01; *n*=5). A single band at 40 kDa was detected in the OE (Fig. 3D). An enzymatic deglycosylation protocol was carried out using gill tissues and, following this, only a single band at 29 kDa was observed (Fig. 3E). No bands were present in western blots generated with gill and OE samples incubated with anti-Cldn-10c primary antibody in combination with immunopeptide.

### Immunohistochemistry

#### Claudin-10c in OE

##### Effect of hypersaline conditions

**Fig. 4.**
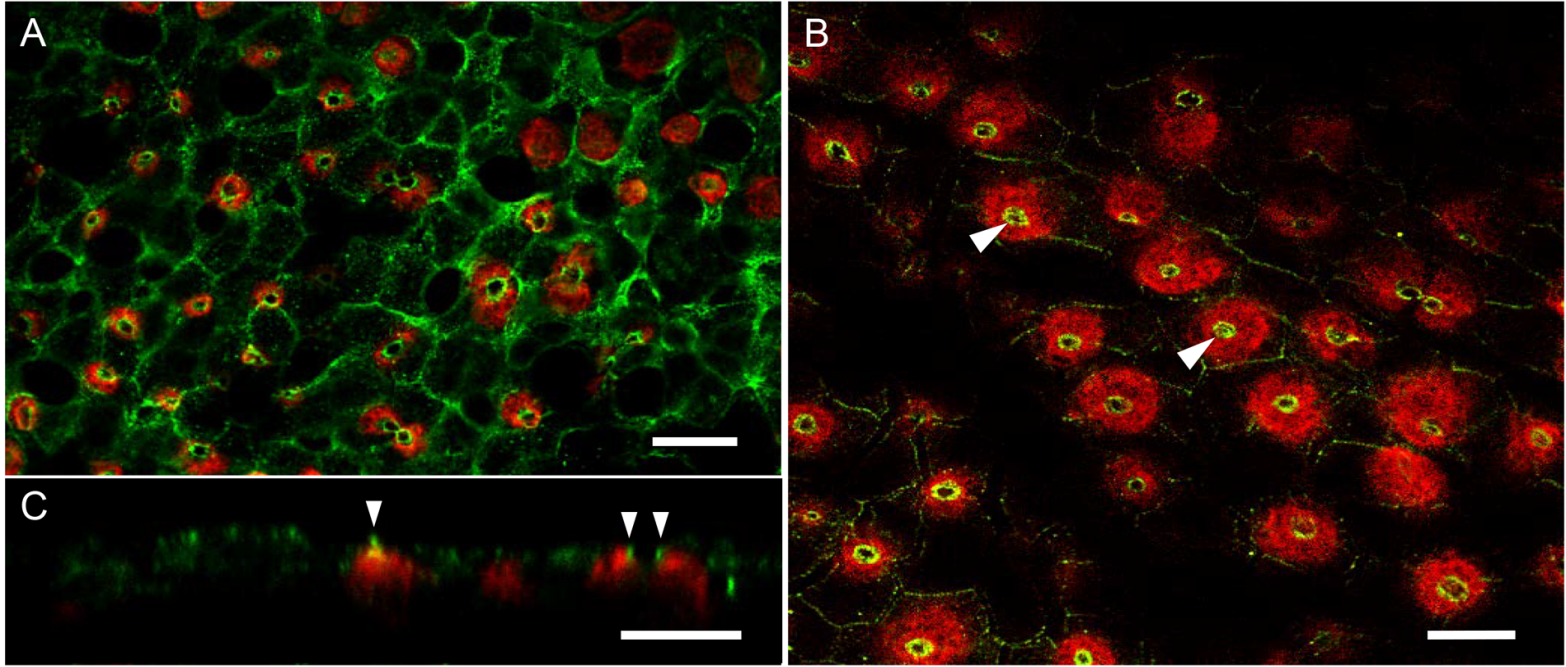
**Immunohistochemistry of mummichog Cldn-10c (green) and Na^+^,K^+^-ATPase (red) in ionocytes of opercular epithelium from mummichogs acclimated to seawater (A; *n*=10) and hypersaline (2SW, B; *n*=4).** (A) XY scan at the level of ionocyte apical crypts with ring-like Cldn-10c fluorescence around ionocyte crypt and some fluorescence in tight junctions between pavement cells. (B) XY scan at the same level as A with intense fluorescent rings (arrowheads) in ionocyte crypts and enlarged hyperplastic cells. Panel C shows an XZ focal plane. Scale bars: 10 µm.

Cldn-10c immunofluorescence in SW acclimated fish OE consistently appeared in the apical crypts of ionocytes as incomplete or complete rings (Fig. 4A) slightly (1–2 µm) above (Fig. 4B) the immunofluorescence of Na^+^,K^+^-ATPase, which served as a marker for mitochondrion-rich ionocytes (*n*=10 epithelial preparations on day 10 of 2SW acclimation). TJs between pavement cells (PVCs) were often observed to be variably positive for Cldn-10c (Fig. 4A). Deeper into the epithelium (10–14 µm), there regularly appeared Cldn-10c immunopositive ionocytes (i.e. as defined by the parallel presence of Na^+^, K^+^-ATPase immunoreactivity) also appearing as ring-like shapes. This intraepithelial staining we inferred as unerupted ionocytes (data not shown). Exposure to 2SW (*n*=4, exposures 5, 11, 17 and 25 days in 2SW) did not alter the localization of Cldn-10c and Na^+^,K^+^-ATPase immunofluorescence (i.e. Cldn-10c immunofluorescence was still in the apical crypts of ionocytes) and there was weakly positive signal in the TJs between PVCs. In addition, ionocyte cell size, indicated by the Na^+^,K^+^-ATPase positive area, was markedly larger (Fig. 4B, 2SW exposure 17 days). The width of the Cldn-10c positive rings appeared wider, typically 0.5 µm, compared to 0.3 µm in SW, often the rings were double lines (arrow in Fig. 4C) and the signal stronger in the 2SW ionocytes. The estimated volume of ionocytes in SW was 576±34.3 µm^3^ (*n*=100 cells from ten images) but after exposure to 2SW for 11, 17 and 25 days, ionocyte volume was significantly (*P*<0.001, unpaired two tailed *t*-test) larger at 1492±88.7 µm^3^(*n*=100 cells from ten images). For the latter, images from observations at day 11, 17 and 25 were pooled because a steady-state condition was achieved at these time points. There was no significant change (*P*=0.3549) in the overall ionocyte density (number of ionocytes per unit area); in SW 49.3±5.1 cells per 0.015 mm^2^ versus 2SW at 40.3±8.1 cells per 0.015 mm^2^ (*n*=10 areas of 0.015 mm^2^ per sample).

##### Proximity of Cldn-10c relative to CFTR and actin

**Fig. 5.**
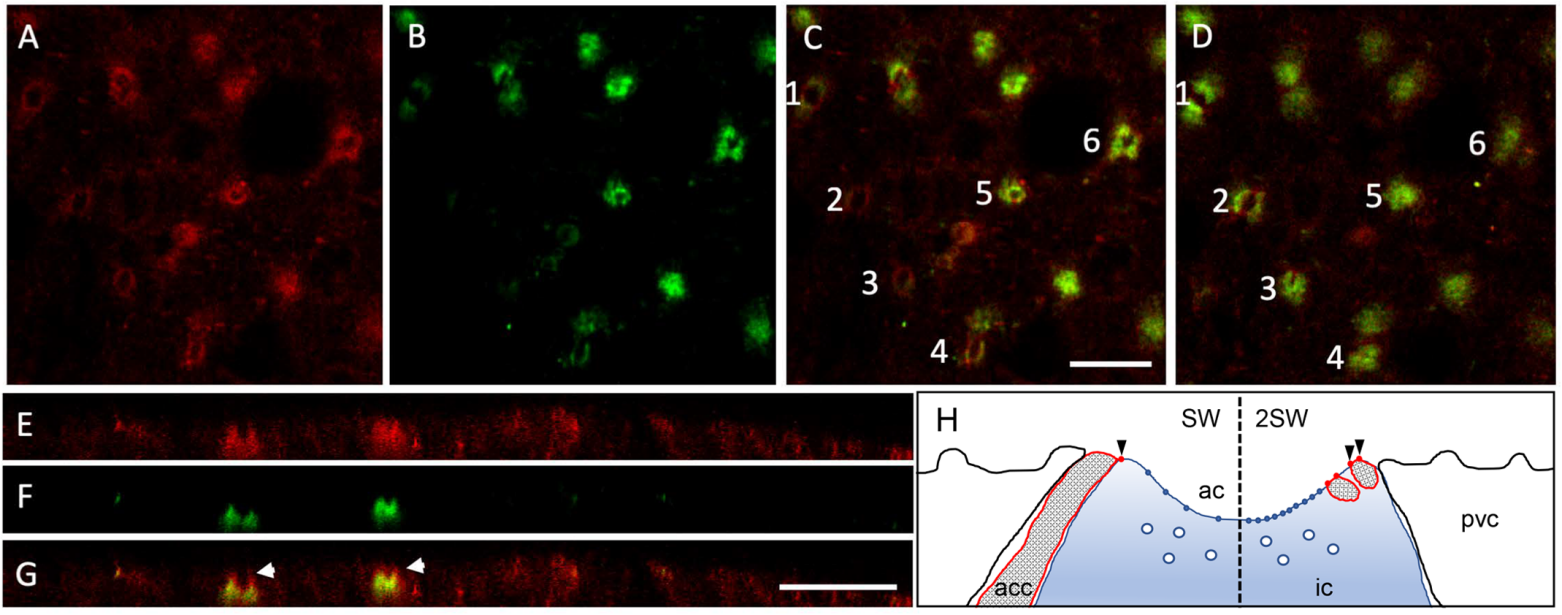
**Seawater opercular epithelium ionocytes.** (A) Red: Cldn-10c, (B) green: CFTR anion channels. (C) merged signals; near the surface of the epithelium with red rings apparent in superficial cells (cells 1, 2, 3 and 4) but in slightly deeper sections (cells 5 and 6) rings stain for CFTR also, yielding colocalization. (D) Same frame and cells but 2 µm deeper: note that in cells 1, 2, 3 and 4, Cldn-10c is colocalized with CFTR and that cells 5 and 6 have closed appearance CFTR (indicating the bottom of the cup-shaped apical crypt) and no red stain. E, F and G are XZ scans through ionocytes (E) Cldn-10c (F) CFTR and (G) Merge. Note that Cldn-10c immunofluorescence extends above the zone of colocalization with CFTR, arrowheads. *n*=10. Scale bars: 10 µm. Panel H is a summary illustration of apical portion and apical crypt (ac) of an ionocyte (ic) with pavement cells (pvc), accessory cell (acc) as well as acc processes with Cldn-10c pores (red dots and arrowheads) positioned somewhat above the apical membrane-resident CFTR anion channels (blue dots), with more of the acc processes, Cldn pores and CFTR channels in 2SW (right), compared to SW control conditions (left). Sub-apical vesicles (white circles in ionocyte sub-apical region, commonly seen in TEM) are the presumed location of sub-apical CFTR.

**Fig. 6.**
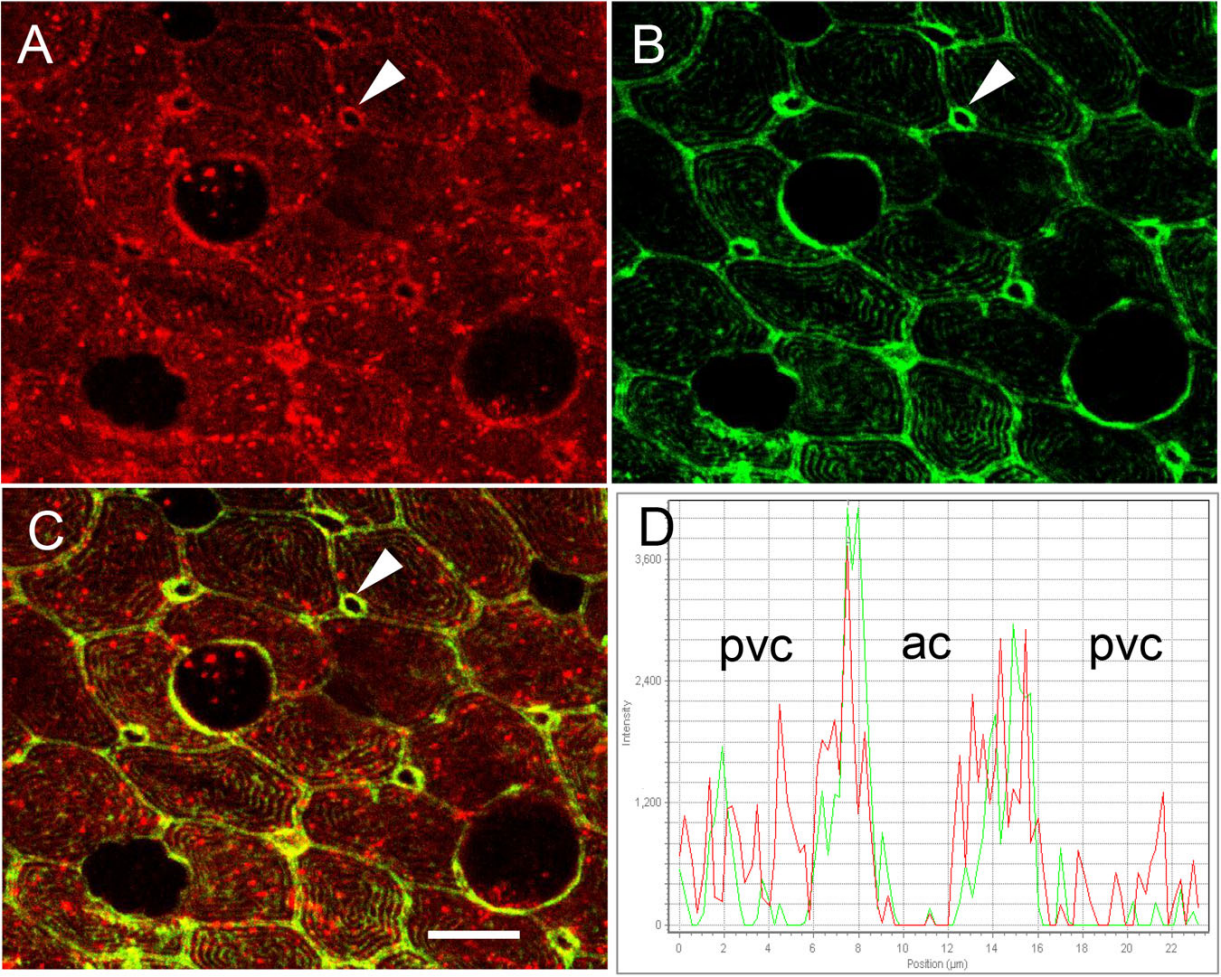
**Immunohistochemistry of ionocytes in opercular epithelium of SW mummichog showing distribution of Cldn-10c and JLA20 (F-actin).** Cldn-10c (A; red) and actin (B; green) are localized in the apical crypts of ionocytes (white arrowheads highlight one of seven ionocytes in the images) and in the pavement cells. (C) Actin colocalizes with Cldn-10c in the apical crypt of ionocytes and in the TJs of the pavement cells (yellow in merged image; *N*=6). (D) A line scan across an apical crypt shows both green and red fluorescence overlapping at the edges of the apical crypt. Scale bar: 10 µm.

In the SW OE, Cldn-10c was detected in apical crypts (Fig. 5A–G) as seen in images collected using the red channel for Cldn-10c and the green channel to localize CFTR. CFTR distribution is cup-like, down the sides of the apical crypts and across the bottom of the crypts, whereas, Cldn-10c occurred slightly (1–2 µm) higher along the margins of the apical crypt than CFTR and was colocalized with CFTR along the sides of the apical crypts but was absent from the bottom of the crypt (XY scans Fig. 5A–D and XZ scans, Fig. 5E–G). Fig. 5H diagrammatically summarizes the findings of more cation-permeable claudin-10c and more anion-conductive CFTR in 2SW than in SW controls and the close relative positions of the two proteins in the ionocytes. Cldn-10c was also detected in the PVCs of the OE but most prominently in the apical crypts of ionocytes (arrowhead) (Fig. 6A). Actin immunofluorescence was present in the microridges on the upper surface of pavement cells and in the rings surrounding ionocyte apical crypts (Fig. 6B). Cldn-10c colocalized considerably with actin in the apical crypts and in the TJs between pavement cells (Fig. 6C,D).

**Fig. 7.**
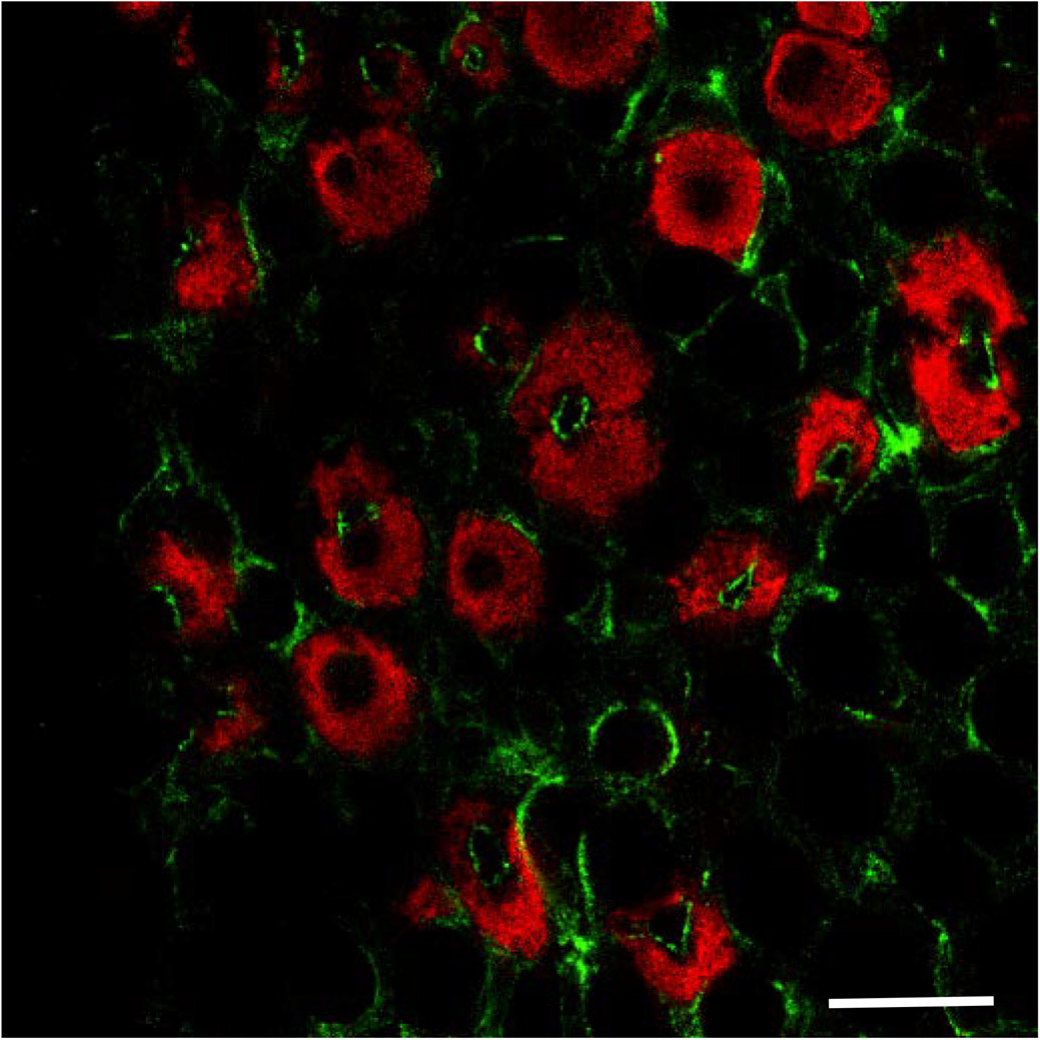
**Cldn-10c immunofluorescence in FW acclimated mummichog opercular epithelium ionocytes (*n*=4 animals).** Green is anti-Cldn-10c; red is α-5 anti Na^+^, K^+^-ATPase. Image was collected 10 µm below the surface of the epithelium, showing some unexposed ionocytes with Cldn immunofluorescence and pairs of enlarged ionocytes. There was no positive Cldn immunofluorescence at the plane of the surface of the epithelium. Scale bar: 20 µm.

FW acclimated mummichog opercular epithelia had some ionocytes below the surface in the OE that appeared to stain for Cldn-10c, localized to the apical crypts (Fig. 7). However, many of Na^+^, K^+^-ATPase positive ionocytes were negative for Cldn-10c (Fig. 7). Consistent with FW living, the Cldn-10c positive ionocytes were not exposed to the surface of the epithelium and instead were buried 10 µm or more in the epithelium.

#### Claudin-10c in gill

**Fig. 8.**
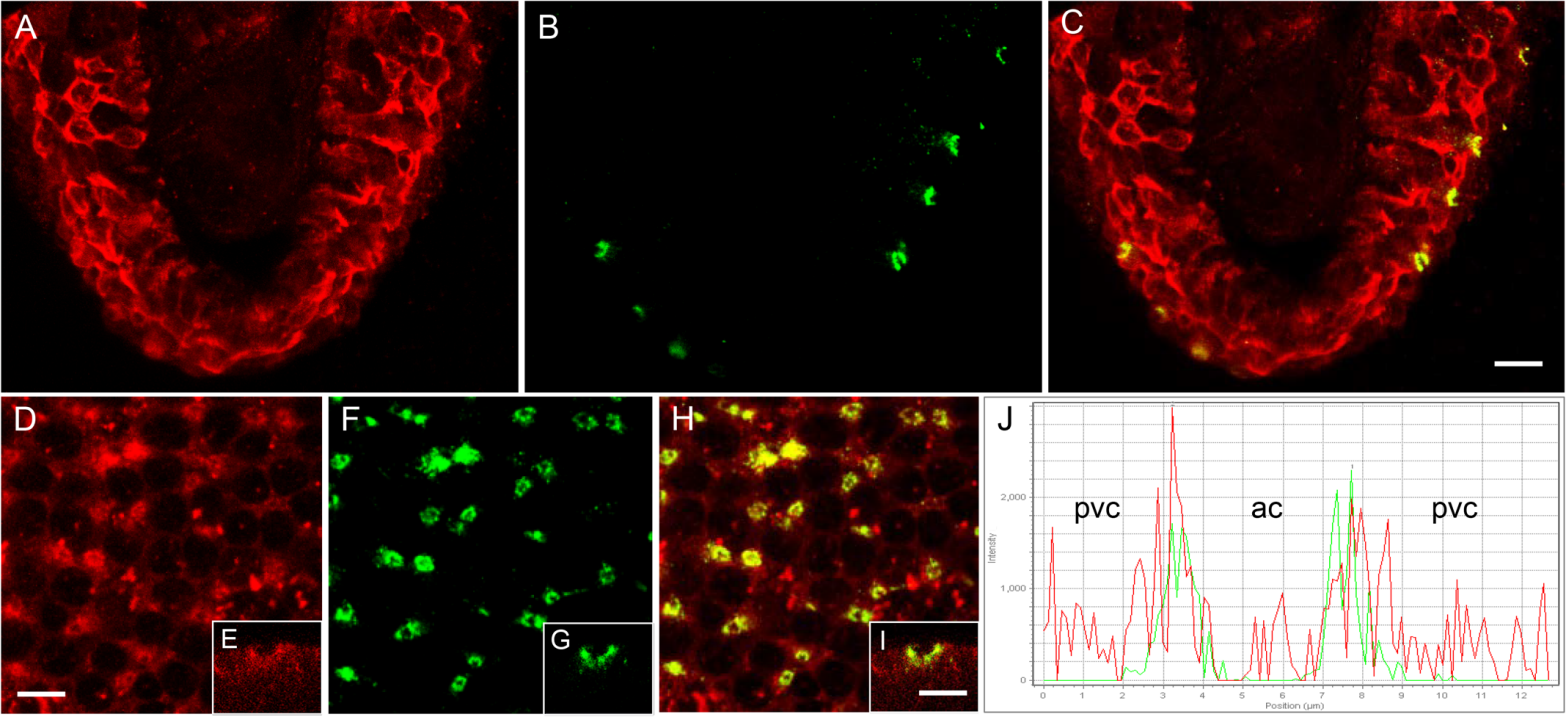
**Immunohistochemistry of SW mummichog gill cross-sections (A**–**C) and longitudinal sections across the surface of the posterior gill filament (D**–**I) showing the distribution of Cldn-10c and CFTR anion channels.** (A) Cldn-10c (red) is localized to the margins of the apical crypt (ac) of ionocytes and to a lesser extent in the pavement cells (pvc). (B) CFTR (green) is localized solely across the apical crypts of ionocytes. (C) Colocalization of Cldn-10c and CFTR in the apical crypt of ionocytes (yellow). In the longitudinal gill sections, Cldn-10c (D, XZ side view E) and CFTR (F, XZ side view G) colocalize in the apical crypt (H, I XZ side view) shown here by the overlapping red and green fluorescence in the line scan graph (J). (*n*=6) ac: apical crypt, pvc: pavement cells. Scale bar: 10 µm for A,B,C,D,F,H. Scale bar: 5 µm for E,G, I.

The cellular localization and distribution of Cldn-10c in SW fish gill filaments were similar to that observed in OE ionocytes. Specifically, some immunofluorescence was observed in pavement cells (Fig. 8A), but it was at a high intensity in apical crypts of ionocytes. CFTR immunofluorescence was present in ionocyte apical crypts (Fig. 8B) where CFTR colocalized with Cldn-10c (Fig. 8C). In a different plane, distribution of Cldn-10c was further evident in the pavement cells and with prevalent immunofluorescence in the ionocyte apical crypts (Fig. 8D,E). CFTR (Fig. 8F,G) was obvious in the apical crypt rings of ionocytes, as was colocalization of the two proteins (Fig. 8H,I); the colocalization was also confirmed by line scan graph (Fig. 8J).

## DISCUSSION

### Overview

The current study provides a first look at organ-specific profiles of *cldn-10* paralogs in the mummichog, *F. heteroclitus* and, with respect to externally exposed ion-secreting epithelia, shows that the gill and OE exhibit an almost identical *cldn-10* paralog profile. Because recent evidence supports the view that *cldn-10* paralogs are essential components of the paracellular Na^+^ secretory pathway of the SW-residing mummichog gill (Marshall et al., 2018), this observation underscores the OE as an important and appropriate surrogate model of gill epithelium. Furthermore, salinity-induced changes in the transcript and protein abundance of *cldn-10* paralogs in gill and OE (i.e. changes occurring following acclimation from SW to 2SW) mirrored one another, thus supporting the view that these organs exhibit functionally consistent paracellular properties using the same TJ proteins. This idea is additionally supported by observations of the cellular and sub-cellular localization of Cldn-10c protein in OE and gill, using a custom antibody to the carboxy terminus. Specifically, Cldn-10c is a hypersaline responsive paralog at the transcriptional level (Marshall et al., 2018) and, in this study, was shown to exhibit parallel changes in protein abundance. Furthermore, Cldn-10c exhibited robust localization in gill and OE ionocytes in which co-immunofluorescence with CFTR further demonstrated that Cldn-10c in this species is associated with intercellular junctions adjoining apical crypts. Finally, the current study detected a heavier anti-Cldn-10c reactive band in both the gill and OE that was eliminated by deglycosylation enzymes. This band was more intense in the OE and in both gill and OE under hypersaline conditions. Our findings suggest a mechanism that could assist in the maintenance of elevated functional Cldn-10c under conditions of extreme salinity (i.e. a hypersaline environment) as protein glycosylation has been associated with environmentally stressful circumstances (e.g. Ernst et al., 1994; Kazemi et al., 2010). This also suggests that under natural conditions, the molecular physiology of the OE TJ complex may be better prepared for rapid exposure to hypersaline conditions.

### Organ-specific *cldn-10* paralog and *cftr* mRNA expression/abundance and salinity response

The TJ protein Cldn-10 has been documented in multiple forms in teleost fishes and these are now quite broadly reported to exhibit restricted organ-specific distribution patterns that are often dominated by the gill (Loh et al., 2004; Tipsmark et al., 2008; Bui and Kelly, 2014; Kolosov et al., 2014). In addition, Cldn-10 proteins (or the genes that encode them) in the gill epithelium are also well-documented to respond to alterations in environmental salinity, such that they typically increase following transfer of euryhaline fishes from FW to SW (e.g. Tipsmark et al., 2008; Bui et al., 2010; Bui and Kelly, 2014; Marshall et al., 2018) or from SW to 2SW, as was observed more recently (Marshall et al., 2018). The current study shows that *cldn-10* transcript distribution patterns in the mummichog were found to occur predominantly in the gill as well as in the OE. While the observations of gill *cldn-10* abundance are largely confirmatory, an OE *cldn* profile has yet to be reported for any fish species. Nevertheless, the presence of TJ strands in mummichog OE have been well illustrated by transmission electron microscopy (e.g. Karnaky, 1991), and the OE is an established surrogate model for studying the ion transport properties of the marine fish gill (Degnan et al., 1977; Marshall et al., 1997; Marshall and Grosell, 2006). Therefore, observations of *cldn-10* paralogs in the OE that qualitatively and quantitatively match those of the gill provide evidence that the molecular components, which facilitate paracellular Na^+^ secretion across the OE are, if not the same, very similar to those of the gill epithelium. In turn, these observations also underscore the suitability of the OE as a model for studying marine fish ion transport. Indeed, further emphasizing this conclusion is the response of OE *cldn-10* paralogs following acclimation of animals from SW to 2SW, which closely align with the response of *cldn-10* paralogs in the gill. That is, *cldn-10c* and *-10f* were the *cldn-10* paralogs that exhibited robust and clear-cut elevations in mRNA abundance in the gill and OE following 2SW acclimation in this study and these *cldn-10* paralogs also exhibited robust and sustained elevations in gill tissues following acclimation to 2SW in a previous study (Marshall et al., 2018).

Because the OE is a flat epithelial sheet that is often referred to as ‘skin’, it is important to note that the molecular physiology of OE *cldn-10*s does not resemble the epidermal epithelium. Specifically, only one *cldn-10* paralog (*cldn-10c*) was found to be expressed in the general integument of the mummichog, and in this organ *cldn-10c* transcript abundance was markedly lower than *cldn-10c* transcript abundance in the gill and OE. This indicates that the molecular physiology of OE TJs resembles the gill more than the skin. The presence of *cldn-10c* in the skin would be consistent with the low level Cldn-10c immunofluorescence we observed in branchial junctions between PVCs. More specifically, because branchial PVCs as well as epidermal cells are connected by multi-stranded junctions that exhibit low permeability (Chasiotis et al., 2012; Kolosov et al., 2013; Sardet et al., 1979), apparently some copies of pore-forming claudins can exist in these locations without coming together to form organized single-stranded ion pores. Nevertheless, *cldn-10c* transcript abundance in the skin of the mummichog in this study did increase in response to 2SW acclimation. At this stage, it can be suggested that increased mummichog skin *cldn-10c* is unlikely to contribute to overall salt and water balance, because *cldn-10c* abundance is low and the ion-transport capacity of the cutanenous epithelium of most teleost fishes, beyond the larval stage, is limited. Indeed, comparable observations and a similar conclusion was drawn by Bui and Kelly (2014) with respect to low levels of *cldn-10* transcript abundance in the puffer fish skin versus the gill epithelium. Nevertheless, a physiological role for select Cldn-10 proteins seems likely in the skin, but will require further study.

Transcript-encoding *cldn-10* paralogs were also present in the brain and eye (*cldn-10c*, *-10d*, and *-10f*) as well as the kidney (*cldn-10f*), liver and posterior intestine (*cldn-10d*). Transcript abundance in these organs was considerably lower than that observed in the gill and OE, and in no case did transcript abundance alter following exposure to 2SW. However, these observations do suggest that Cldn-10 proteins have a physiological role to play in barrier tissues that are not directly exposed to the external environment in the way that the gill and OE are. But we are far from understanding all the complexities of Cldn-10 protein involvement in teleost fish homeostasis. Transcript encoding *cldn-10* in the central nervous system illustrate this point, as *cldn-10* paralogs have been reported in the nervous tissue of several teleost species (see Kolosov et al., 2013), but species-specific differences in which form dominates seem to occur. For example, in the mummichog brain *cldn-10d* and *cldn-10f* are predominant, whereas *cldn-10e* was not detected. This contrasts with the rainbow trout brain where *cldn-10e* is present but *cldn-10c* and *-10d* are absent (Kolosov et al., 2014). This difference between species was further seen in *cldn-10* paralog expression in ocular tissues, and contrasting both are observations derived from *Fugu rubripes*, where no *cldn-10* expression was detected in the brain, while *cldn-10b* and *-10c* were detected in the eye (Loh et al., 2004). Taken together with observations of selective *cldn-10* presence in the liver, kidney and posterior intestine, organ-specific *cldn-10* distribution appears to highlight the significance and importance of diverse strategies in the maintenance of physiological barriers and ion balance.

### Cldn-10c in the gill and OE

Transcriptional changes in branchial *cldn-10*s during and following salinity acclimation have been documented in several teleost species, with increases in teleost Cldn-10 protein abundance documented in the spotted green pufferfish (Bui and Kelly, 2014). In the current study, acclimation to 2SW resulted in elevated Cldn-10c in the mummichog gill and OE. An anti-Cldn-10c custom antibody detected a 29 kDa band in the gill, consistent with the MW prediction of a 255 residue sequence (XP_012728690). In addition, a ∼40 kDa band in both the gill and the OE was also detected throughout this study. The 40 kDa band exhibited similar immunoreactivity as the predicted sequence, which could suggest a peptide that has a similar epitope to the Cldn-10c carboxy terminus sequence (against which the primary antibody was raised), potentially leading to non-specific interactions. However, BLAST searches of the *F. heteroclitus* genome failed to reveal any full-length matches and only two hits at 43% similarity (GABA receptor type 5 and Frizzled-10, neither of which are associated with epithelial ion transport) and no matches for the highly immunogenic last eight residues (KTASNVYV-COOH). Further analysis suggests an alternative hypothesis, that the MW shift of Cldn-10c may be a product of post-translational modification. *In silico* analysis of Cldn-10c sequence revealed 22 potential O-glycosylation sites (NetOGlyc 4.0; Technical University of Denmark). An estimate of 22 glycosylation sites would shift the MW by 4.8 kDa with each glycosylation site presumably modified with a 220 Da glycoside. Whereas simple glycosylation in serine and threonine glycosidation is typical, there exists a possibility where the glycoside-units may be further elongated by glycan repeats (Nakada, 2014; Ogawa et al., 2018). The 40 kDa immunoreactive band was absent after glycosidase incubation, indicating that the observed 40 kDa band may be a heavily glycosylated protein. Whereas the current study has not explored potential interplay between phosphorylation and glycosidation that may alter the integrity of TJ components (Butt et al., 2012), an *in silico* prediction of YinYang sites using YinOYang 1.2 (Gupta and Brunak, 2002) revealed seven potential YinYang sites in the mummichog *cldn-10c* sequence, five near the carboxy terminus. Hence further investigation of mummichog *cldn-10c* regulation is warranted. Nevertheless, a MW shift towards 40 kDa that is greater in the OE samples was detected, to a point where analyses of the 29 kDa band in the OE is delimited due to experimental resolution. Experimental evidence for the glycosylation of Cldn TJ proteins is scarce, and most glycosylation information on Cldns is based on sequence information. Here we conclude that Cldn-10c can be glycosylated in the mummichog gill and OE epithelium, with a predominant form in the OE that seems likely to have functional significance.

Glycosylation is a post-translational modification that is associated with sub-cellular functions that include signal-transduction, protein localization, and protein stability (for review, see Moremen et al., 2012) and there is evidence to support protein glycosylation as an intrinsic cellular response to environmental stressors that enhances the stability of proteins (Ernst et al., 1994; Watanabe et al., 2004; Kazemi et al., 2010). Notably, these observations include glycosylation of CFTR, an ion-transporting protein in a salt-stressed ion-transporting epithelium (see Ernst et al., 1994). Taken together with observations in this study, it can be suggested that glycosylation of Cldn-10c in branchial tissues may be a mechanism that allows externally exposed mummichog ion-regulating epithelia to cope with the heightened environmental challenge of hypersaline conditions. Furthermore, because the putative glycosylated form is dominant in the OE, this may mean that the OE is better equipped for a rapid exposure to hypersaline conditions. This idea, however, will require further exploration.

The increase in Cldn-10c abundance underlines the importance of its potential contribution in the formation of TJ pores that drive cation permeability. The high transcript versus comparatively modest (but still significant) increase in Cldn-10c protein abundance suggests a potentially high turnover rate of *cldn-10c* mRNA. The dual increases in *cldn-10c* and *cftr* transcripts (which mirror one another) and the resulting Cldn-10c abundance further strengthens the idea that Cldn-10c plays an important role in modulating epithelium permeability in response to hypersaline surroundings.

### Claudin-10 immunohistochemistry and salinity response

In OE and gill there is a close-fitting association of Cldn-10c immunofluorescence in the TJs of Na^+^-K^+^-ATPase-enriched ionocytes that have CFTR in the apical membrane. Importantly, immunogen peptide was able to block Cldn-10c immunofluorescence in SW opercular epithelia and omission of the primary antibody also produced negligible fluorescence. The close association of Cldn-10c and CFTR in the OE (fixed in methanol-DMSO) and in the gill (fixed in paraformaldehyde) demonstrates the robust binding ability of the custom Cldn-10c antibody and also supports the use of the OE as a model for ionocyte function in the marine teleost gill. The claudin immunofluorescence in XZ scans was clearly located more apically than Na^+^, K^+^-ATPase, consistent with a TJ position. Cldn-10c immunofluorescence was also colocalized with CFTR in part, along the sides of the apical crypts in XZ scans, but Cldn-10c was absent from the bottom of the cup-shaped apical membrane, where only CFTR was present. We infer that the Cldn-10c was not in the apical membrane but rather, in the TJ complex.

Actin is present near epithelial TJs and immunohistochemistry revealed that Cldn-10c and F-actin were co-localized in the apical crypt area and to a lesser extent in the TJ between pavement cells, consistent with actin involvement in TJ maintenance and a TJ localization for Cldn-10c. Cldn-10c localization in association with gill and OE ionocytes was similar to that previously observed in human conjunctival epithelium (Yoshida et al., 2009) in that there was punctate distribution of human CLDN-10 in conjunctiva associated with specialized transport cells, rather than a general staining of TJs, as was observed for CLDN-1, -4 and -7. Despite being present in both ionocytes and PVCs of the gill and OE, overall Cldn-10c was enriched in mummichog ionocytes. In the puffer fish gill epithelium, Cldn-10d and -10e were also found to be enriched in ionocytes (Bui and Kelly, 2014), but based on molecular and cell isolation techniques, were proposed to be absent from PVCs (Bui et al., 2010). Similarly, *cldn-10c* and *-10d* were proposed to be absent from gill PVCs of rainbow trout and enriched in ionocytes (Kolosov et al., 2014). But *cldn-10e* transcript was found in both ionocytes and PVCs of rainbow trout, suggesting that species-specific differences in the presence (or absence) of *cldn-10*s in different branchial epithelium cells occurs. Nevertheless, we can conclude that immunohisochemical evidence places Cldn-10c in or in close association with mummichog branchial TJs, especially in the apical crypts of ionocytes and closely associated with important transport proteins CFTR and Na^+^,K^+^-ATPase as well as F-actin associated with TJ complex function.

Hypersaline conditions produced marked hyperplasia of ionocytes in the OE, similar to that previously observed in branchial ionocytes of hypersaline tolerant fishes (Gonzalez, 2012), and an apparent increase in the thickness of the Cldn-10c immunofluorescent rings, consistent with a need to increase ion transport rate in the higher salinity. We have observed a significant increase in opercular epithelium transepithelial potential in hypersaline conditions *in vitro*, such that the electrical driving force for Na^+^ extrusion rises from +35–40 mV in SW to +45–50 mV in 2SW, accompanied by an increase in epithelial conductance (Marshall et al., 2017) along with elaborations in ionocyte-AC TJs (Cozzi et al., 2015). Ion substitution experiments demonstrated that the isolated OE selects for Na^+^ and against Li^+^, Rb^+^, and Cs^+^ (Marshall et al., 2018), evidence that paracellular Cldn-10 pores are Na^+^-selective. Our results are consistent with those of Ouattara et al. (2009), who observed a marked increase in ionocyte size in tilapia (*Sarotherodon melanotheron*) gill epithelium without an apparent increase in cell density, following exposure to hypersaline conditions. Therefore all evidence suggests that hypersaline conditions enhances Cldn-10c presence in the TJs of ionocytes and increases the cell capacity for ion transport in the form of cation-selective pores that comprise a Na^+^-selective paracellular pathway essential for NaCl secretion.

### Perspective

The current study presents further evidence that Cldn-10 proteins play a key role in regulating salt secretion across the branchial tissues of fishes acclimated to or living in SW or hypersaline conditions. Of particular note is the idea that in the mummichog, Cldn-10 proteins exhibit a functional response to hyperosmotic condition, and that Cldn-10c in this species is an important component in the formation of TJ pores that contribute to the permselectivity properties of branchial epithelia under SW and hypersaline conditions. This most likely contributes to the ability of these fish to accommodate paracellular cation secretion under such extreme conditions. We are also able to conclude that the molecular physiology of genes encoding Cldn-10 proteins in the mummichog exhibit a consistent response to hypersaline conditions in the gill and OE, providing the first evidence that the molecular machinery of the cation-selective TJ complex of the OE and gill epithelium are the same. But it is also clear from this study (and others) that while we can make some generalizations about Cldn-10 TJ proteins, the specific presence and role of different Cldn-10 in branchial epithelia and other barrier tissues in fishes will not be uniform, and this warrants further study. Indeed, future studies that also address the functional significance Cldn glycosylation in fishes will also be of interest.

## MATERIALS AND METHODS

### Animals

Adult mummichogs (*Fundulus heteroclitus* Linnaeus, 1766) of both sexes (7–15 g mass) were trapped in Ogdens Pond, Antigonish County, Nova Scotia, Canada in June and transported in coolers containing estuary water to the St. Francis Xavier University Animal Care Facility. The fish were placed in full strength SW (32‰) in 450 l recirculating tanks at room temperature (20±1°C) under 12L:12D photoperiod under artificial lighting and were held for several weeks prior to experimentation. Salinity was monitored daily using an YSI Pro2030 (YSI Inc., Yellow Springs, OH, USA) conductivity meter; partial water exchanges were performed at the rate of one-third aquarium volume (same salinity and temperature) per 48 h period. For acclimation to hypersaline conditions, 30 fish were exposed to a flow-through salinity change, first to 45‰ SW for 48 h, then to 60‰ SW (2SW) for 6 days. Fish were sampled at 6 days following transfer to 60 ‰ for RNA and protein isolation, and at days 11, 17, and 25 (post transfer to 60 ‰) for immunohistochemistry. SW was made hypersaline by addition of artificial sea salt (Instant Ocean, Blacksburg VA, USA). For acclimation to FW, fish were directly transferred to dechlorinated Antigonish tap water (0.1 mM NaCl, pH 6.7–6.9) for at least 10 days before use in immunofluorescence experiments. Fish were fed mealworms (*Tenebrio molitor*) 3 days a week and Nutrafin flakes (R.C. Hagen, Montreal, Quebec, Canada) twice daily, so that each fish consumed 1.0 g of food per 100 g of body weight per day. Fish were euthanized by single pithing followed by decapitation. For organ/tissue-specific profiling of *cldn-10* paralogs, brain, eye, gill, OE, heart, liver, kidney, posterior intestine (i.e. last third of total intestine length), skin (general body surface) and flank muscle were rapidly dissected, flash frozen in liquid N_2_ and stored at −80°C until further analysis. For immunohistochemistry, OE and gill filaments were dissected in and/or rinsed with modified Cortland's saline (composition in mmol l^−1^: NaCl 159.9, KCl 2.55, CaCl_2_ 1.56, MgSO_4_ 0.93, NaH_2_PO_4_ 2.97, NaHCO_3_ 17.85, and glucose 5.55, bubbled with a 99% O_2_/1.0% CO_2_ gas mixture, pH 7.7–7.8, osmolality 317 mOsm kg^−1^) prior to further processing and fixation (see below). Animal care and treatment occurred under approval 16-003-R2 by St. Francis Xavier University Animal Care Committee, following Canadian Council on Animal Care guidelines.

### Total RNA isolation and quantitative real-time PCR analyses

Samples for gene transcript expression profiles were homogenized in TRIzol reagent (Invitrogen Canada Inc., Burlington, ON, Canada) for total RNA isolation according to the manufacturer's protocol. RNA pellets were resuspended in an appropriate volume of RNase-free water and quantified using a Nanodrop 2000 spectrophotometer (Thermo Fisher Scientific, Waltham, MA, USA) with sample quality determined by A260/A280 ratio. A fixed quantity of total RNA from each sample (2 µg) was treated with DNase I (Amplification Grade; Invitrogen Canada Inc.). First-strand synthesis was then carried out using SuperScript III™ reverse transcriptase and Oligo (dT)_12-18_ primers (Invitrogen Canada Inc.).

Quantitative-PCR (qPCR) experiments were carried out using iQ SYBR Green Supermix (Bio-Rad Laboratories, Inc., Mississauga, ON, Canada) in a CFX-96 Real-time System (Bio-Rad, Hercules, CA, USA) under the following conditions: 95°C for 10 min, 40 cycles of 95°C for 30 s, 60°C for 60 s, read every cycle, 59°C to 95°C for melt curve, read every 0.5°C. PCR primers for *cldns-10c*, -*10d*, -*10e*, -*10f*, *cftr*, and *18S RNA* were as previously described by Marshall et al. (2018). Standard curves were generated with stock mummichog gill cDNA in each qPCR assay as an internal control of each qPCR run. *18S RNA* was used as a reference gene and prior to analysis, *18S RNA* usage was validated statistically by comparing raw *Cq* values between organs/treatments to ensure no statistically significant changes occurred (*P*=0.882). Primer efficiency values averaged at 91.6% with specific values as follows; *18S*=83.6%, *cldn-10c*=83.52%, *cldn-10d*=96.6%, *cldn-10e*=105.5%, *cldn-10f*=90.4%, and *cftr*=90.0%. Transcript abundance calculations and primer efficiency corrections were as reported by Marshall et al. (2018). Organ-specific transcript abundance of *cldn-10*s and *cftr* are reported using *18S RNA* as a reference gene and expressed as fold change relative to levels in the SW fish gill. Transcript abundance of *cldn-10*s and *cftr* in gill, OE and skin following 2SW acclimation are reported using *18S RNA* as a reference gene and for each organ, are expressed as fold change relative to SW levels.

### Antibodies

The primary antibody used for detection of mummichog CFTR was mouse monoclonal anti-human CFTR (MAB25031, clone 24-1, R&D Systems, Minneapolis, USA) with the epitope at the carboxy terminus, a zone that is conserved in mummichog to human (Singer et al., 1998, 2008) and therefore is selective for this protein (Marshall et al., 2002). Custom synthesized anti-*F. heteroclitus* Cldn-10c affinity purified rabbit polyclonal antibody (developed by GenScript USA Inc., Piscataway, NJ, USA) was used for detection of mummichog Cldn-10c carboxy terminus; the immunogen sequence was: CISNTTRKTASNVYV. Cldn-10c was selected for antibody development because this paralog exhibited increased transcript abundance in gill tissue following acclimation from SW to hypersaline conditions (Marshall et al., 2018). F-actin immunofluorescence was detected using JLA20 mouse monoclonal antibody (Marshall et al., 2017) and α-5 mouse monoclonal antibody to the alpha (catalytic) subunit of chicken Na^+^,K^+^-ATPase, which were obtained from Developmental Studies Hybridoma Bank (DSHB, University of Iowa, Iowa City, USA). Secondary antibodies used for immunofluorescence microscopy were goat anti-rabbit polyclonal immunoglobulin-G conjugated with Alexa Fluor 488 (A11001, Life Technologies Inc., Eugene, OR, USA) and goat anti-rabbit polyclonal immunoglobulin-G conjugated with DyLight 549 (111-505-003, Jackson ImmunoResearch Laboratories Inc., West Grove, PA, USA).

### Immunoblots

Protein samples for immunoblotting of Cldn-10c were prepared from the phenol-ethanol fraction of the total RNA isolation solution according to the manufacturer's protocol. The fractions were first washed with anhydrous ethanol to remove remaining nucleic acids in the solution, and protein samples were precipitated with 100% isopropanol followed by three washes (20 min each) in 0.3 M guanidine hydrochloride dissolved in 95% ethanol. Protein pellets were then washed once (20 min) with anhydrous ethanol prior to resuspension in 1% sodium dodecyl sulfate (1% SDS). Protein concentration of each sample was determined using a Micro bicinchoninic (BCA) Protein Assay Kit (Thermo Scientific, Rockford, IL, USA) according to the manufacturer's instructions for the Microplate Procedure and samples were read using a Multiskan spectrophotometer (Thermo Fisher Scientific, Nepean, ON, Canada). For protein separation, tricine-SDS-PAGE electrophoresis was carried out with a 4% stacking gel and 10% separating gel in a Mini-PROTEAN Tetra Cell apparatus (Bio-Rad Laboratories, Inc., Mississauga, ON, Canada) with a Tris-Tricine buffer (25 mM Tris, 25 mM Tricine, 0.05% (w/v) SDS; Bioshop Canada, Burlington, ON, Canada). Identical amount of protein samples were treated with a sample buffer (Tris 60 mM; SDS 2%; glycerol 10%; Bromophenol Blue 0.0025%; 3% β-mercaptoethanol), heated (70°C, 15 min), and allowed to migrate at a constant voltage of 150 V.

Protein samples were blotted onto a PVDF membrane (0.45 µm pore size; Merck Millipore) with a chilled wet-transfer unit (Amersham Biosciences; 150 mA; 80 min). After transfer, membranes were briefly rinsed in Tris-buffered saline with 0.1% Tween-20 (TBS-T; 20 mM Tris, 150 mM NaCl, pH 7.6) and blocked with a blocking solution (immunoblot blocking solution; 5% skim milk in TBS-T adjusted to 250 mM NaCl) for 1 h at room temperature. The membrane was then incubated at 4°C with Cldn-10c primary antibody solution (0.75 µg Cldn-10c primary antibody/ml immunoblot blocking solution) overnight. Immunogen peptide blocking was performed by pre-incubating the Cldn-10c primary with peptide immunogen (1.5 µg Cldn-10c peptide immunogen/ml Cldn-10c primary antibody solution) overnight. An HRP conjugate secondary antibody (goat anti-rabbit; Bio-Rad Laboratories, Hercules, CA, USA) and an ECL substrate (Pierce Biotechnology, Rockford, IL, USA) were used for detection of Cldn-10c on PVDF membranes. Blots were imaged using DiaFilm Autoradiography Film (Diamed Lab Supplies Inc., Mississauga, ON, Canada) and digitized. Blots were then treated with an acid stripping buffer, re-probed with an internal loading control (F-actin, JLA20, DHSB) and imaged. Densitometry analysis of immunoreactive bands was carried out in Image Lab 6.0 software (Ver. 6.0.0 build 26; Bio-Rad Laboratories, Inc., Hercules, CA, USA). When two Cldn-10c immunoreactive bands appeared on the same blot (i.e. at 29 and 40 kDa for gill tissues), the densitometric area examined encompassed both bands, and overall data was reported. In addition, densitometry of 29 kDa (gill) as well as 40 kDa (gill or opercular epithelium) bands were examined individually by examining areas that independently encompassed those bands.

### Deglycosylation of Cldn-10c

Deglycosylation of gill and OE protein samples was carried out with an Enzymatic Protein Deglycosylation Kit (EDEGLY, Millipore-Sigma). Protein samples from the gill and OE (100 µg in each case) were denatured (100°C, 5 min) and cooled to room temperature prior to addition of deglycosylation enzymes including PNGase F, O-Glycosidase, α-2(3,6,8,9)-Neuraminidase, β-1→4-Galactosidase, and β-N-Acetylglucosaminidase. The reaction mixture was incubated in a 37°C water bath for 6 h. Samples were used immediately for immunoblots after incubation. A negative-control experiment was performed to verify the deglycosylation kit buffer and detergent components have minimal effect on the mobility shift and intensity of the bands in the gill and OE samples.

### OE Immunohistochemistry

OE from FW, SW and 2SW acclimated fish were dissected in Cortland's saline, excluding the underlying dermal chromatophore layers and whole mounts pinned to modeler's sheet wax (700-079, Rio Grande, Albuquerque, NM, USA). Preparations were rinsed three times in rinsing buffer (TPBS) comprising 0.1% bovine serum albumin (BSA) with 0.05% Tween 20 in phosphate-buffered saline (PBS, composition in mmol l^−1^: NaCl 137, KCl 2.7, Na_2_HPO_4_ 4.3, and KH_2_PO_4_ 1.4 at pH 7.4). OE were fixed for 2 h at −20°C in 80% methanol/20% dimethyl sulfoxide (DMSO), then rinsed in TPBS and immersed in a blocking solution (immunohistochemistry blocking solution) made with 5% normal goat serum, 0.1% BSA, 0.2% NaN_3_ in TPBS, pH 7.4 for 1 h at 24°C in the dark. The OE were then incubated in the primary antibodies Cldn-10c and JLA20 or CFTR (8 µg ml^−1^ in immunohistochemistry blocking solution) at 4°C overnight. Following three (5 min) rinses in PBS, the OE were exposed to the secondary antibodies (8 µg ml^−1^ in immunohistochemistry blocking solution) for 4 h at room temperature in the dark. After three final rinses in PBS, the OE were covered in a mounting medium (Fluoroshield™, Sigma-Aldrich, St. Louis, MO, USA) and slides were viewed in a single blind fashion. Negative controls were performed by immunogen peptide blocking; Cldn-10c immunogen peptide was pre-incubated with the primary antibody in immunohistochemistry blocking solution, then added to the OE. Subsequent handling of negative controls was performed identically as above. Images were collected with a laser scanning confocal microscope (FV300, Olympus, Markham, ON, Canada). In each OE, fields were randomly selected and *z*-stack series were collected using a 40× water (N.A. 1.15W) or 60× water objective (N.A. 1.20W), zoom of 3.0 and XYZ optical sections at increments of 0.50 µm (Marshall et al., 2017).

### Gill histology and immunohistochemistry

Gill arches from SW fish were dissected, rinsed with Cortland's saline and fixed in 4% paraformaldehyde (PFA) pH 7.4, at 4°C for 24 h. Fixation in 4% PFA provides mechanical support during vibratome sectioning. Following fixation, the gills were rinsed three times in phosphate-buffered saline (PBS; composition in mmol l^−1^: NaCl 137, KCl 2.7, Na_2_HPO_4_ 4.3, KH_2_PO_4_ 1.4, pH 7.4). The gill filaments were removed and embedded in 3% w/v agarose in PBS and allowed to set for 20 min at 4°C. Filaments were sectioned by vibratome (Vibratome^®^ Series 1000, Technical Products International, Inc., St. Louis, MO, USA) into 200 μm thick cross-sections and longitudinal sections (Barnes et al., 2014). The sections were immersed in immunohistochemistry blocking solution for 1 h at 24°C (in the dark) then incubated with primary antibodies (8 μg ml^−1^), anti-CFTR and anti-Cldn-10c, in immunohistochemistry blocking solution overnight at 4°C. Sections were then rinsed three times in TPBS rinsing buffer and exposed to the secondary antibody goat anti-mouse IgG complexed with Alexa Fluor 488 (8 μg ml^−1^, Life Technologies Inc., Eugene, OR, USA) and goat anti-rabbit IgG DyLight 549 (8 μg ml^−1^, Vector Laboratories, Burlingame, CA, USA) in immunohistochemistry blocking solution for 4 h at 24°C (in the dark). After three final rinses in TPBS rinsing buffer the sections were mounted in histology mounting medium (Fluoroshield™, Sigma-Aldrich). Negative (null) controls were performed by omitting the primary antibodies. Slides were viewed and images were collected with a laser scanning confocal microscope (FV300, Olympus). Optical section stacks were collected for final figures.

### Statistics

All data are expressed as mean±s.e.m. (*n*) where *n* represents the number of fish within the group. A Student's *t*-test was used to evaluate the effect of salinity on a target organ. Significant differences (*P*<0.05) are denoted with an asterisk (*). Ionocyte cell volume was calculated as oblate ellipsoids from XYZ confocal scans, *n*=100 cells from 10 XYZ images, *n*=4 animals per group, and statistical differences detected using unpaired two-tailed *t*-tests. All statistical analyses were performed using SigmaPlot for Windows Version 14.0 (Build 14.0.3.192; Systat Software, Inc., San Jose, CA, USA).

## Supplementary Material

Supplementary information
