# Supplementary material for: Mummichog gill and operculum exhibit functionally consistent claudin-10 paralog profiles and Claudin-10c hypersaline response
Source: Biol Open. 2021 Jul 26;10(7):bio058868. doi: 10.1242/bio.058868 (PMC8351317; doi:10.1242/bio.058868)
Supplement: Supplementary information [file biolopen-10-058868-s1.pdf]

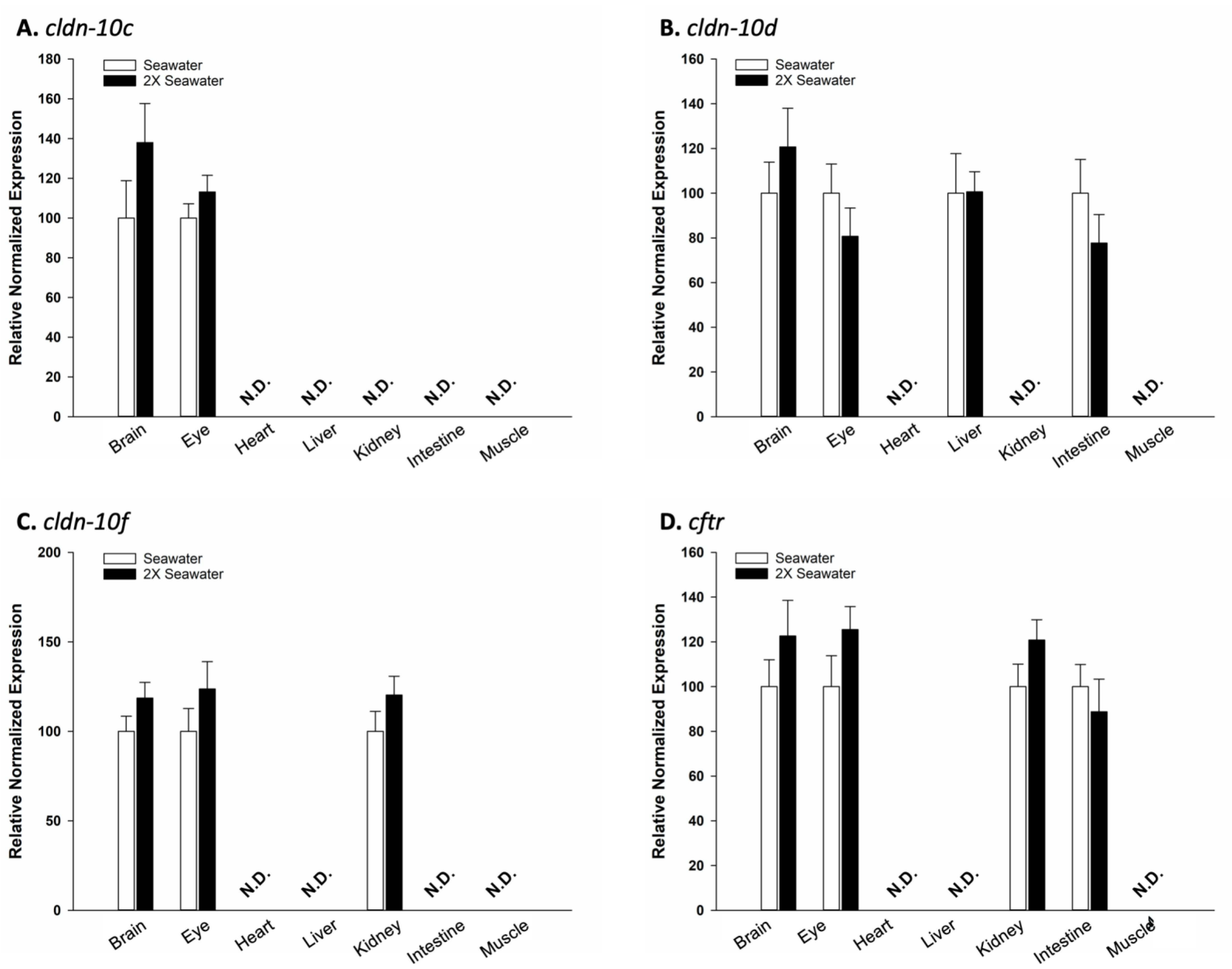

Fig. S1. Transcript abundance of claudin (cldn) (A) -*10c*, (B) -*10d*, (C) -*10f*, and (D) cystic fibrosis transmembrane conductance regulator (*cftr*) in mummichog brain, eye, heart, liver, kidney, intestine, and muscle following acclimation of animals from seawater (SW; open bars) to hypersaline conditions (2xSW; black bars). Transcript abundance was normalized using 18S RNA in the target tissue and 2SW transcript abundance in each organ was expressed relative to SW assigned a value of 100. All data are expressed as mean values±s.e.m. (n=5). N.D., transcript not detected.

Biology Open • Supplementary information

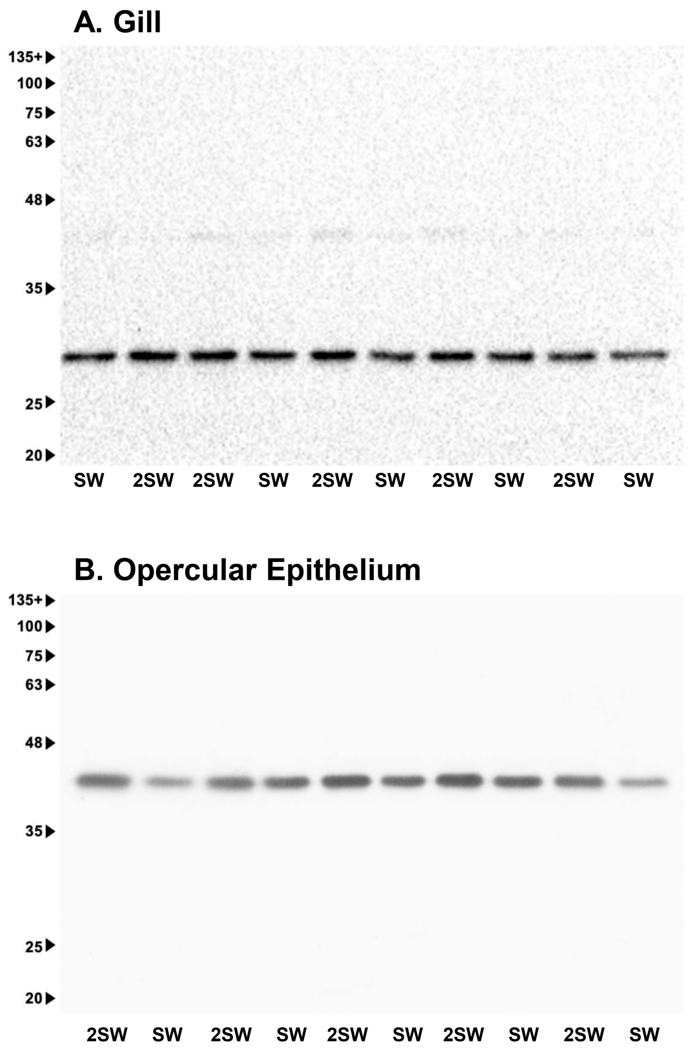

Fig. S2. Immunoblots of claudin-10c (Cldn-10c) using (A) gill and (B) opercular epithelium tissues from fish acclimated to seawater (SW) or hypersaline conditions (2SW).

Biology Open • Supplementary information
